# An algorithm for automated closure during assembly

**DOI:** 10.1186/1471-2105-11-457

**Published:** 2010-09-10

**Authors:** Sergey Koren, Jason R Miller, Brian P Walenz, Granger Sutton

**Affiliations:** 1 The J. Craig Venter Institute, 9712 Medical Center Drive, Rockville MD 20850, USA

## Abstract

**Background:**

Finishing is the process of improving the quality and utility of draft genome sequences generated by shotgun sequencing and computational assembly. Finishing can involve targeted sequencing. Finishing reads may be incorporated by manual or automated means. One automated method uses targeted addition by local re-assembly of gap regions. An obvious alternative uses *de novo *assembly of all the reads.

**Results:**

A procedure called the bounding read algorithm was developed for assembly of shotgun reads plus finishing reads and their constraints, targeting repeat regions. The algorithm was implemented within the Celera Assembler software and its pyrosequencing-specific variant, CABOG. The implementation was tested on Sanger and pyrosequencing data from six genomes. The bounding read assemblies were compared to assemblies from two other methods on the same data. The algorithm generates improved assemblies of repeat regions, closing and tiling some gaps while degrading none.

**Conclusions:**

The algorithm is useful for small-genome automated finishing projects. Our implementation is available as open-source from http://wgs-assembler.sourceforge.net under the GNU Public License.

## Background

The shotgun method generates reads randomly in high volumes by Sanger and next-generation sequencing platforms. Whole-genome shotgun assembly (WGA) is the process of constructing a draft assembly of a genome from whole-genome shotgun reads (WGS). WGA software constructs a read layout by inference from shared sequence between reads and constraints between pairs of reads from the same DNA fragment (paired-ends). The randomness of WGS can be exploited in software by adopting uniformity of read coverage as an objective function to be maximized by the assembly. For instance, the Celera Assembler software [1] invokes the A-stat coverage statistic to assign lower confidence to higher-coverage mini-assemblies. The Velvet software [2] invokes low-coverage to trim branches of its de Bruijn graph.

Finishing is the process of improving the quality and utility of a draft genome sequence. Finishing aims to fill gaps between contigs, enlarge contigs, or provide deeper coverage for the contigs in the draft. Some finishing is accomplished without sequencing by manually editing an automatically generated draft. Most finishing requires additional sequence referred to as finishing reads. Finishing reads derive from PCR, primer walking, transposon bombing, shotgun of individual clones and other techniques. See [3] for a review.

Intuitively, a PCR experiment provides evidence that reads generated from within the PCR product should be assembled between instances of the primer sequences. The set of finishing reads derived from an individual amplicon, or clone, have a co-location requirement. End-reads from amplicons or clones provide the boundaries between which the finishing reads should assemble.

Manual inspection and placement of finishing reads is expensive [4], even when assisted by software. The widely-used Consed package [5] provides assembly editing functionality through a graphical user interface. Dupfinisher [6] automates several finishing procedures, including homology-based search to identify repeats in the draft assembly. As an example, Consed and Dupfinisher were invoked during the finishing stage of the *Pedobacter heparinus *genome project [7]. After 44K WGS reads had been assembled with phrap http://www.phrap.org, Dupfinisher corrected mis-assemblies. Then, with 1897 finishing reads, Consed and manual editing were used to close gaps and improve quality. Both of these methods are *a posteriori*, running after an assembly has been generated. They require users to specify a gap for each finishing read. In contrast, we introduce an algorithm for placing finishing reads within the context of a WGA.

Our method generates a *de novo *assembly in one process that integrates the WGS and finishing reads. We only require the identifiers for the end-reads from the amplicons or clones, potentially improving usability. The algorithm uses the sets of finishing reads and placement bounds for each set to incorporate finishing reads during the assembly process. Our algorithm targets repetitive genomic regions, seeking to close and thicken repeat-induced gaps, as well as to locate repeat copies missed in the initial assembly.

The algorithm was challenged to assemble five prokaryotes and one eukaryotic genome from WGS and finishing reads. The results were compared to assemblies of all the reads input as WGS reads. The results were also compared to an alternate assembly pipeline.

## Results

### Algorithm

#### Input

The input has three components: WGS reads, finishing reads, and bounding constraints. The reads may include paired-end reads, such that any given read pair consists of two WGS reads or two finishing reads. The bounding constraint is usually a paired end thought to span the target of the finishing reactions, based on a WGS assembly. Alternately, it could be any two reads whose sequence encompasses the PCR primers that generated the template for the finishing reads, or the PCR primers themselves. Formally, given the set of bounded finishing reads *F*, and WGS reads *W*, the finishing reads and bounding constraints must satisfy the following conditions:

• For each finishing read, *f *∈ *F*, there exists at most one pair of reads, *f_a _*∈ *W *and *f_b _*∈ *W *s.t. *f_a _*≠ *f_b_*, *f *≠ *f_a_*, and *f *≠ *f_b _*(referred to as the bounding constraint for f)

• For each bounding constraint, (*f_a_*, *f_b_*), there exists a non-empty set of finishing reads, $\bar{\left(f_{a},f_{b}\right)}=\{f_{1}\ldots f_{n}\}$

• For any two bounding constraints, (*f_a_*, *f_b_*) and (*g_a_*, *g_b_*), the intersection $\bar{\left(f_{a,}f_{b}\right)}\cap \bar{\left(g_{a},g_{b}\right)}=\{\}$

#### Assemble the reads

The algorithm uses the hierarchy of overlaps, unitigs, contigs, and scaffolds as used by Celera Assembler [1]. The WGS and finishing reads are processed to detect pair-wise overlaps. Reads and overlaps are compressed into unitigs, also called chunks [8]. The unitigs are low-risk assemblies consistent with nearly all of the detectable pair-wise read overlaps. Unitigs that are deemed repetitive, *V*, are not trusted. All the others, *U*, are trusted. The trusted unitigs are assembled into contigs and scaffolds using detected pair-wise overlaps and paired-end constraints. Untrusted unitigs are incorporated last. The contigs represent contiguous assembly. The scaffolds consist of contigs separated by gaps whose length is estimated from paired-end constraints.

#### Fill the gaps

The algorithm applies aggressive techniques to fill gaps in scaffolds.

It starts by assigning left over unitigs (including individual reads) to specific gaps. It generates a list *G *of gaps in scaffolds, a list *U *of unique trusted unitigs not placed in scaffolds, and a list *V *of all untrusted repeat unitigs. For each unitig *u *∈ {*U *⋃ *V*}, and a gap *g *∈ *G*, define a placement score *Place*(*u*, *g*) = *P *+ *B *where *P *= (number of WGS reads in *u *whose paired-end constraint would be satisfied by placement in gap *g*) and *B *= (number of finishing reads in *u *whose bounding constraint would be satisfied by placement in gap *g*). A paired-end constraint is satisfied when one read is within *u *while the second is not and the placement of unitig *u *in gap *g *matches the expected orientation and distance of the paired-end library. A bounding constraint is satisfied when the bounding reads (*f_a_*, *f_b_*) are both placed in a scaffold and span only gap *g*. For each unitig *u *∈ *U*, assign it one gap that maximizes *Place*(*u*, *g*) > 0, if one exists. For each unitig *u *∈ *V*, assign it all gaps for which *Place*(*u*, *g*) >*Q *for some threshold *Q*.

Once the unitigs are placed within gaps, we apply a miniature assembly process. Estimate each gap's length from spanning paired-end constraints. For each gap, construct a graph whose nodes are unitigs assigned to the gap. Add a node for both of the contigs that bound the gap. Add an edge for every unitig pair, or unitig/contig pair, that shares paired-ends or has a detectable sequence overlap. Search for any path through the graph that satisfies the size estimate and visits each node at most once. If such a path is found, the gap is filled with unitigs from the path. If no path spans the gap, the algorithm will settle for less: adding a unitig to each contig end so as to reduce the size of the remaining gap. This gap-filling step runs twice: first with unassembled unitigs from *U*, then with all unassembled unitigs from *U *and *V*.

**Figure 1:**
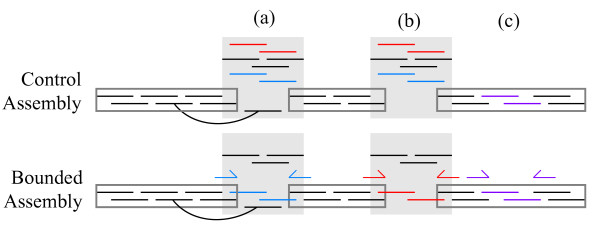
**Use of finishing reads**. Two algorithms for assembling shotgun reads and finishing reads. The control treats both read types equally. The bounded algorithm attempts to assemble finishing reads consistently with their bounding constraints. For each algorithm, the figure shows its construction of a scaffold from contigs (rectangles) with 2X in shotgun reads (black lines). Each finishing read (colored line) has a corresponding pair of PCR primer sites (arrows of same color). External to the scaffold is a unitig (grey area) deemed repetitive due to high coverage. **(a) **A mate pair constraint (curve) localizes one read and the unitig to this gap. Nevertheless, the control algorithm cannot tile this gap with reads. The bounded algorithm localizes two finishing reads by their primer sites. The bounded algorithm does tile the gap with reads, enabling a more accurate consensus sequence. **(b) **The control cannot localize the unitig or any reads to this gap. It does not close the gap. The bounded algorithm localizes the unitig by finishing reads and their primer sites. It tiles the gap with finishing reads from the unitig. **(c) **Both algorithms assemble finishing reads from a gap that is not a genomic repeat. In our data sets, most finishing reads fit gaps of this type.

For all unitigs in scaffolds, a multiple sequence alignment of reads is determined. Repeat unitigs, *V*, can have zero, one, or many placements in the assembly, but their reads can have at most one placement (Figure 1a). For each unitig *u *∈ *V *with at least one placement in a scaffold, every read *r *∈ *u *is assigned a specific location, if possible. Read *r *is placed only if there is unambiguous support from the placement of *u*, and the mate of *r*, if any, or the bounding constraint of *r*, if any.

### Implementation

In order to exploit the maturity of WGS assembly software, we implemented the algorithm inside an existing package, Celera Assembler. Originally designed for Sanger data [1], the software now incorporates alternate modules into a pipeline called CABOG [9] specifically for data from the 454 Life Sciences http://www.454.com next-generation sequencing platform.

The bounding read behaviour is offered as a run-time option in Celera Assembler. When turned on, it executes as part of the scaffold module, specifically during the gap filling stage called "rocks and stones" [1]. To search the set of unitigs that could fill a gap, Celera Assembler uses a breadth first search algorithm. As always, Celera Assembler calculates the scaffold consensus sequence given the hierarchical layout of contigs, unitigs, and reads.

Where the new code needs to partition the unitigs into *U *and *V*, it re-uses Celera Assembler's partition, which is based on empirical observation of the distribution of unitig coverage levels and calculation of the A-stat log-odds ratio [1].

The implementation allows that some constraints are inherently unsatisfiable. Constraints where the bounds span multiple gaps or are not part of the assembly are considered unsatisfiable and the current implementation does not use them. The threshold *Q*, used to place unitigs from *V *in scaffold gaps, is calculated automatically at run time for each unitig. The threshold defaults to the number of unsatisfiable constraints. Intuitively, this value of *Q *requires more evidence for a unitig placement than against.

### Evaluation

#### Data sets

Six genomes were selected for testing (Additional file 1: Table S1). The genomes include five bacteria and one protozoan. All six WGS data sets include Sanger sequence. Three are predominantly pyrosequencing data. All six finishing read sets include Sanger reads from selected WGS clones.

#### Test of the algorithm

Contiguity statistics, such as N50 are course-grained and mask the improvements that a small numbers of finishing reads can provide. Therefore, we introduce a concept of candidate regions and we measure how many candidates are improved. The candidates are genome repeats for which we have finishing reads and bounding constraints. Potential improvements consisted of closing a gap (Figure 1b), adding read coverage across a repeat, and fixing the consensus sequence (Figure 1a). The candidates are exclusive of non-repeat regions (Figure 1c) for which the control algorithm is sufficient. Improvement was measured by comparing the bounded read assemblies against control assemblies. The controls used the same software and reads without the bounding constraints.

#### Comparison to alternate assemblers

Dupfinisher is a pipeline for assembly, repeat identification, finishing read generation, and re-assembly. It is integrated with phrap, BLAST, Consed, and Autofinish [10]. It was not feasible or meaningful to snap Dupfinisher into our assembly pipeline or to snap our assembler into its pipeline. Also, it was not possible to compare published experiments. Papers that describe Dupfinisher report combined gains of the software plus manual editing (*e.g. *[7]) or present results on projects [6] for which we could not obtain finishing reads.

The Newbler *de novo *assembly software http://www.454.com is designed specifically for pyrosequencing reads alone or in combination with Sanger data. It also supports incremental assembly, whereby additional reads are added to a previous assembly result. The incremental assembly feature can be used to add finishing reads to assemblies of WGS reads. Newbler was tested on three genomes for which we had pyrosequencing reads.

## Results

The bounded algorithm closes 52 candidate regions, previously having either no sequence or no read coverage. The control, by definition, closes 0 and the alternate pipeline closes 4. This large gain can be attributed to incorporating more finishing reads. The algorithm incorporates 75.10% of finishing reads. That is ≈54% more than control and ≈42% more than the alternate. The assemblies show a gain of 0.09 ± 0.09% in percent of the genome with >1X read coverage versus control. Full details of the per-genome assembly improvements are in Table 1. The bounded algorithm also fixes 63 consensus bases while introducing only 3 incorrect calls in comparison to the control versus reference. Details are available in Table 2.

**Table 1:** Results using the bounded read placement algorithm.

				Placed finishing reads			Gaps closed		
Species	Candidate gaps	# Bounds	# Finishing reads	Control	Bounded	Alternate	Control	Bounded	Alternate
					
*E. coli *O157:H7	14 (1)	56	128	26	92	N/A	0	11	N/A
*S. enterica*	2 (0)	18	33	14	23	N/A	0	1	N/A
*B. mallei*	9 (0)	23	40	4	27	N/A	0	4	N/A
*I. multifiliis*	11 (2)	14	21	3	21	17	0	6	4
*E. coli *K12	49 (2)	23	60	12	49	11	0	29	0
*C. amycolatum*	4 (0)	3	3	0	2	0	0	1	0
Total	89 (5)	137	285	59	214	28	0	52	4

Comparison of three algorithms. Control uses finishing reads like WGS reads. Bounded uses finishing reads with placement constraints. Alternate uses finishing reads in a second round of assembly without constraints. Candidate gaps include both regions in the control assembly between finishing constraints with zero coverage and a consensus sequence derived from a repeat unitig or no consensus sequence in the control assembly. The parentheses indicate the number of gaps with no consensus sequence in the control assembly. The gap and spanning constraint are not necessarily 1-to-1. Bounds: The total number of bounding constraints that span the repeat gap or were not satisfied in both control and bounded assemblies. Finishing reads: The total number of finishing reads generated for the bounds in the table. Placed finishing reads: The total number of finishing reads placed in the assembly by each of the assembly algorithms. Gaps closed: The number of gaps closed by filling in missing consensus sequence or by tiling repeat instances with reads. By definition, the control assembly always closes 0 gaps. The bounded assembly joins were verified by alignment to finished reference, where available.

**Table 2:** Consensus quality of the bounded read placement algorithm.

Species	# consensus differences	True positives	False positives
*E. coli *O157:H7	14	14	0
*S. enterica*	5	5	0
*B. mallei*	47	44	3
*I. multifiliis*	N/A	N/A	N/A
*E. coli *K12	N/A	N/A	N/A
*C. amycolatum*	N/A	N/A	N/A

Total	66	63	3

Performance of the same three algorithms described in Table 1. Number of consensus differences: The total number of bases in consensus that are different between the bounded and control assemblies versus reference. True positive: Number of consensus base changes that are supported by the reference. False positive: The number of consensus base changes that differ from the reference. The finishing reads used for *E. coli K12 *did not come from the same strain as the reference. We cannot validate whether a consensus discrepancy between an assembly and the reference is due to assembly error or to strain-level differences. Consensus quality could not be measured on the two genomes that lack a reference.

In three of the six genomes, the algorithm places additional copies of repeat unitigs, filling in missing consensus sequence. As a consequence, ten contigs are merged. Due to the coarseness of the contig metrics, the bounded assemblies show improvement (fewer contigs and higher N50) in one genome but no change on the rest. In two genomes, the merging involved one contig greater than 2Kbp and one smaller than 2Kbp and is therefore not reflected in Table 3.

**Table 3:** Results using contig metrics for bounding read placement algorithm.

	**Contig count**			**Contig N50**			**Contig bases**		
**Species**	**Control**	**Bounded**	**Alternate**	**Control**	**Bounded**	**Alternate**	**Control**	**Bounded**	**Alternate**
			
*E. coli *O157:H7	6	5	NA	2,315,032	4,484,293	NA	5,656,811	5,661,119	NA
*S. enterica*	6	6	NA	3,620,140	3,620,144	NA	4,813,438	4,813,442	NA
*B. mallei*	19	19	NA	424,003	424,003	NA	5,835,215	5,834,616	NA
*I. multifiliis*	4,273	4,273	5,765	12,070	12,070	11,444	37,616,884	37,616,884	47,976,992
*E. coli *K12	313	314	387	27,255	27,255	16,838	4,679,711	4,679,711	4,441,778
*C. amycolatum*	26	26	38	307,040	307,040	152,524	2,525,388	2,525,392	2,507,351

Performance of the same three algorithms described in Table 1. Contig count: number of contigs whose consensus is at least 2Kbp. Contig bases: sum of consensus lengths for contigs at least 2Kbp long.

The assemblies of *E. coli *O157:H7 were examined and compared to the available reference. The bounded assembly was confirmed, having eight alignments of 99% identity over 99% length of the assembly covering 99% of the reference. By the same measure, the control assembly had nine alignments. Inspection revealed two high-coverage unitigs each placed six times in the control and seven times in the bounded. Together, the two unitigs make up a seventh repeat instance that was missing from the control. The repeat, which we characterized by NCBI BLAST [11] as an rRNA operon, is known to occur seven times in the wild-type genome [12]. Other joins in the bounded assembly were also verified by comparison to the reference, indicating no mis-assembly (Additional file 1)

The bounding read algorithm placed a majority of the finishing reads available for each genome. On no genome did the algorithm close all the gaps or tile all the candidate regions. The algorithm closed 52 out of 89 possible candidates, this may be due to limitations of the finishing read set rather than the algorithm as no assembly was able to close all candidates. In summary, the bounding read algorithm consistently augmented repeat resolution and gap closure by finishing read placement and improved the consensus.

## Discussion

We implemented our algorithm within the Celera Assembler software for whole-genome shotgun (WGS) assembly. The implementation placed more finishing reads than two alternate methods: *de novo *assembly of WGS reads and finishing reads together (our control), or by adding finishing reads to the initial assembly (with Newbler). This result was not surprising since only our algorithm exploited the finishing read placement constraint data associated with finishing reads.

All of our test data sets included some Sanger WGS reads. Future genome projects are unlikely to present Sanger WGS data due to the lower cost of high-throughput, next-generation sequencing (NGS). Such projects will require clone-free finishing reads generated from genomic template. In this case, each amplicon's end reads can serve as bounds for the other reads derived from that amplicon. Thus, our approach should apply to 100% NGS WGS data sets.

We have presented a novel algorithm for automated re-assembly to exploit finishing reads and placement constraints. Our implementation out-performed two other automated approaches on real data. An alternate approach to finishing, relying on NGS data to correct assembly errors, shows average gains of 0.16 ± 0.15% apart from a single outlier with 6.73% gain [4]. By comparison, our algorithm achieves a gain of 0.09 ± 0.09% through the careful use of existing finishing data, without relying on any additional sequencing. Both methods are valuable to correctly assemble the final pieces of a genome and demonstrate the difficulty involved.

## Conclusions

The finishing process has rate-limiting manual components. Here we demonstrate automation of one finishing component, the careful placement of finishing reads whose position is known relative to other reads. We described the Bounding read algorithm that could be incorporated in a 4-part finishing pipeline: WGS reads are assembled with an assembler; the assembly is scanned for low-quality regions and gaps; finishing reads are generated to target each region; the WGS and finishing reads are re-assembled with the bounding read assembly algorithm.

Earlier approaches to automated finishing use *a posteriori *methods that add finishing reads to assembled contigs. Dupfinisher was the first. Newbler's iterative assembly method demonstrates another. Our approach incorporates finishing reads *a priori *in a *de novo *assembly with the WGS reads. The finishing reads are exploited throughout the assembly construction, possibly generating a different result than the WGS-only assembly. Additionally, our algorithm can identify new instances of recognized repeats and tile reads across them. The algorithm outperformed two alternate methods, filling more gaps, placing more reads, and improving consensus. Our algorithm is a valuable tool to assist the automation and improvement of genome finishing projects.

## Methods

### Reads

The bacterium *Escherichia coli *O157:H7 str. EC4115 was sequenced with Sanger chemistry and is deposited at the NCBI Trace Archive. The reference [GenBank: CP001163], [GenBank: CP001165], [GenBank: CP001164] consists of two circular plasmids and a circular genome of 94,644, 37,452, and 5,572,075 bases respectively.

The bacterium *Escherichia coli *K12 substr. MG1655 was sequenced using 454. The WGS data is available through the Short Read Archive [SRA:SRA001028]. The reference [GenBank: NC_000913] consists of one circular genome of 4,639,675 bases.

The bacterium *Salmonella enterica *subsp. enterica serovar Schwarzengrund str. CVM19633 was sequenced with Sanger chemistry and is deposited at the NCBI Trace Archive. The reference [GenBank: CP001125], [GenBank: CP001126], and [GenBank: CP001127] consists of two circular plasmids and a circular genome of 110,227, 4,585, and 4,709,075 bases respectively.

The bacterium *Burkholderia mallei *NCTC 10247 was sequenced using Sanger chemistry and deposited at the NCBI Trace Archive. The finishing reads are also available from the NCBI Trace Archive. The reference [GenBank: CP000548], and [GenBank: CP000547] consists of two circular chromosomes of 3,495,687 and 2,352,693 bases respectively.

The bacterium *Corynebacterium amycolatum *SK46 HMP033 was sequenced using 454 and Sanger. The WGS data is available through the NCBI Trace Archive and the Short Read Archive [SRA:SRR005142].

The ciliate protozoan *Ichthyophthirius multifiliis *G5 was sequenced using 454 and Sanger. [The data is scheduled to be deposited in the SRA and the NCBI Trace Archive and is available by contacting the authors] The 454 reads were generated by the GS FLX Titanium pyrosequencing platform. They were processed with Celera Assembler to remove duplicates, detect linker, and split paired ends.

For comparison with Dupfinisher, NCBI trace archive was unsuccessfully searched for reads with trace_type_code other than "WGS" belonging to *Methanospirillum hungatei *JF-1, *Rhodoferax ferriducens *DSM 15236, or *Shewanella baltica *OS155.

### Finishing reads

The finishing reads were obtained from JCVI databases. For finishing reads generated from a clone, the clone-end reads were provided to Celera Assembler as bounding reads. Not all finishing reads had bounding reads.

### Software

Celera Assembler software was run using run-time parameters recommended for each sequencing technology. The Sanger-only assemblies used the unitigger module while the assemblies with 454 data used the BOG module from CABOG. The *I. multifiliis *assembly used a 10% error rate instead of defaults. The specific version is marked with CVS tag WGS_CLOSURE-6_00-BRANCH and will be packaged starting with the 6.1 release. Newbler version 2.3 was used with default run-time parameters as the Alternate pipeline. Dupfinisher was kindly provided by its authors.

### Analysis

Continuity statistics were gathered from each assembly using analysis of the FASTA output files. The gap statistics were gathered from each assembly using scripts for analyzing assembly output. The MUMmer package [13] was used to compare assemblies to the references by running *nucmer --maxmatch*.

To identify candidate gaps to evaluate the control assembly we focus on gaps caused by genomic repeats, both with and without consensus sequence in the control. First, we identify regions of the control assemblies that had zero coverage in reads, a consensus sequence due to placement of a (repeat) unitig, and coverage in the unitig at least twice that of the overall scaffold average (Figure 1a). Separately, we listed gaps that have no consensus sequence in the control assembly (Figure 1b). The assemblies were aligned by using *nucmer --maxmatch *and *show-tiling *was used to look for split contigs in either assembly. We also looked for any gaps that have no consensus sequence in the bounded assembly but do in the control. There were none in our datasets.

The *show-snps *program from the MUMmer package was used to identify SNPs between the reference and both control and bounded assemblies. The matches were first filtered by running *delta-filter -1 *and the results used as input for *show-snps *(with no parameters). Regions where the control assembly had gaps (Ns) in the sequence were not included in SNP counts. The total number of SNPs in the bounded assembly but not the control assembly and vice-versa were tabulated.

## Authors' contributions

SK designed the algorithm, implemented the software, ran the tests, and wrote the manuscript. JRM assisted with test design and manuscript revision. BPW participated in the software development. GS proposed the project and reviewed its progress. All authors read and approved the final manuscript.

## Supplementary Material

Additional file 1 **Supplementary Materials**. Supplementary Materials including comparisons to reference and detailed read composition for each data set. Click here for file
