# Supplementary material for: An algorithm for automated closure during assembly
Source: BMC Bioinformatics. 2010 Sep 10;11:457. doi: 10.1186/1471-2105-11-457 (PMC2945939; doi:10.1186/1471-2105-11-457)
Supplement: Additional file 1 — Supplementary Materials. Supplementary Materials including comparisons to reference and detailed read composition for each data set. [file 1471-2105-11-457-S1.DOC]

Supplement

Koren et al. 2010 “An Algorithm for Automated Closure During Assembly”

## Table S1 - Data Sets.

| Species | Sanger WGS Reads | 454 PE WGS Reads | 454 UP WGS Reads | Features with Bounds | Bounded Finishing Reads |
| --- | --- | --- | --- | --- | --- |
| *E. coli* O157:H7 | 59,749 | 0 | 0 | 516 | 1,041 |
| *E. coli* K12 | 1,032 | 67,910 | 185,682 | 516 | 1,041 |
| *S. enterica* | 51,199 | 0 | 0 | 608 | 891 |
| *B. mallei* | 54,980 | 0 | 0 | 825 | 1,235 |
| *C. amycolatum* | 3,410 | 0 | 193,092 | 37 | 45 |
| *I. multifiliis* | 232,924 | 11,332 | 2,606,681 | 734 | 1,233 |

Sequence contribution from each component data set. Sanger WGS Reads: number of paired or unpaired reads from WGS on a Sanger platform. 454 PE WGS Reads: number of reads from WGS pyrosequencing of a paired-end library, after processing to split linker-positive sequences into 2 reads each. 454 UP WGS Reads: number of reads from WGS pyrosequencing of an unpaired library. Features with Bounds: regions targeted by finishing that present at least one finishing read bounded by two other reads. Bounded Finishing Reads: number of finishing reads provided with a bounding constraint. These reads are the focus of the bounding read algorithm. For *E. coli* K12, a 454 library was used in combination with finishing reads and their bounds generated for the *E. coli* O157:H7 project. WGS: whole-genome shotgun. PE: paired end (counting 2 reads per pair). UP: unpaired read.

## *Comparison to Reference:*

*Comparison to Reference.* The assemblies of *E. coli* O157:H7 were examined and compared to the available reference. Eight alignments of 99% identity over 99% length of the Bounded assembly contigs cover 99% of the *E. coli* genome. Nine alignments of 99% identity over 99% length of the Standard assembly contigs cover 99% of the *E. coli* genome. In both cases, there is a mis-join at the end of one contig due to an inverted copy of the rRNA operon The Standard assembly contained two surrogate (repeat) unitigs, each placed six times. In the Bounded assembly both surrogates were placed seven times. An NCBI BLAST [11] search confirmed that the two surrogates make up the rRNA operon, known to occur seven times in the wild-type genome [12]. In addition, several other surrogates were placed one more time in the Bounded assembly than in the Standard assembly. Thus, the Bounded assembly seems to have a more complete representation of this repeated structure.

The assemblies of *E. coli* K12 were examined and compared to the available reference. A total of 313 alignments of 99% identity over 99% length of the Bounded assembly contigs cover 97% of the *E. coli* K12 genome. A total of 313 alignments of 99% identity over 99% length of the Standard assembly contigs cover 97% of the *E. coli* K12 genome. In this case, the Standard and Bounded assembly has the same representation of the reference genome, consistent with their assembly statistics in Table 2.

The assemblies of *S. enterica* were examined and compared to the available reference. Six alignments of 99% identity over 99% length of the Bounded assembly contigs cover 99% of the *S. enterica* genome. Six alignments of 99% identity over 99% length of the Standard assembly contigs cover 99% of the *S. enterica* genome. Once again, the Standard and Bounded assembly has the same representation of the reference genome.

The assemblies of *B. mallei* were examined and compared to the available reference. Nineteen alignments of 99% identity over 99% length of the Bounded assembly contigs cover 99% of the *B. mallei* genome. Nineteen alignments of 99% identity over 99% length over 99% length of the Standard assembly contigs cover 94% of the *B. mallei* genome.
